# Voltage-Gated Ion Channels Are Transcriptional Targets of Sox10 during Oligodendrocyte Development

**DOI:** 10.3390/cells13131159

**Published:** 2024-07-07

**Authors:** Christian Peters, Tim Aberle, Elisabeth Sock, Jessica Brunner, Melanie Küspert, Simone Hillgärtner, Hannah M. Wüst, Michael Wegner

**Affiliations:** Institut für Biochemie, Friedrich-Alexander-Universität Erlangen-Nürnberg, 91054 Erlangen, Germany; christian.peters@fau.de (C.P.); tim.aberle@fau.de (T.A.); elisabeth.sock@fau.de (E.S.); jessica.schindler@fau.de (J.B.); melanie.kuespert@fau.de (M.K.); simone.hillgaertner@fau.de (S.H.)

**Keywords:** glia, myelin, Sox protein, transcriptional control, central nervous system

## Abstract

The transcription factor Sox10 is an important determinant of oligodendroglial identity and influences oligodendroglial development and characteristics at various stages. Starting from RNA-seq data, we here show that the expression of several voltage-gated ion channels with known expression and important function in oligodendroglial cells depends upon Sox10. These include the Na_v_1.1, Ca_v_2.2, K_v_1.1, and Kir4.1 channels. For each of the four encoding genes, we found at least one regulatory region that is activated by Sox10 in vitro and at the same time bound by Sox10 in vivo. Cell-specific deletion of Sox10 in oligodendroglial cells furthermore led to a strong downregulation of all four ion channels in a mouse model and thus in vivo. Our study provides a clear functional link between voltage-gated ion channels and the transcriptional regulatory network in oligodendroglial cells. Furthermore, our study argues that Sox10 exerts at least some of its functions in oligodendrocyte progenitor cells, in myelinating oligodendrocytes, or throughout lineage development via these ion channels. By doing so, we present one way in which oligodendroglial development and properties can be linked to neuronal activity to ensure crosstalk between cell types during the development and function of the central nervous system.

## 1. Introduction

Several voltage-gated ion channels play important roles in oligodendroglial cells. Depending on their temporal expression and exact function, these channels influence the electrophysiological properties of oligodendrocyte progenitor cells (OPCs) and oligodendrocytes with a variety of functional consequences on OPC proliferation, migration, differentiation, myelination, and oligodendrocyte maintenance [1,2,3,4].

Tetrodotoxin-sensitive sodium channels like Na_v_1.1 are, for instance, required in some OPCs for the generation of action potentials and have been implicated in OPC migration and proliferation [5,6,7]. Activation of voltage-gated Ca_v_ channels like the N-type calcium channel Ca_v_2.2 and outward-rectifying K_v_ channels including K_v_1.1 regulate all steps of oligodendrogenesis by promoting proliferation, migration, process extension, differentiation, and myelination [2,8,9,10]. Ca_v_2.2 is still expressed in mature oligodendrocytes and overexpressed in active MS lesions [11,12]. As another prominent example, Kir4.1 inward-rectifying potassium channels in oligodendrocytes are required for extracellular K^+^ removal, particularly in the juxta-axonal space so that their loss from oligodendrocytes impairs K^+^ clearance and leads to seizures, axonal degeneration, and myelin vacuolization in both mice and humans [13,14].

Despite the numerous important functions of voltage-gated ion channels in oligodendroglial cells, little is known about their relationship to the oligodendroglial gene regulatory network that consists of transcription factors, epigenetic regulators, and regulatory RNAs [15,16,17,18]. In particular, it is not known how the network regulates the expression of voltage-gated ion channels. Considering the relevance of voltage-gated ion channels for oligodendroglial development and physiology, it seems reasonable to assume that their expression should be influenced at least in part by the transcription factors that convey identity to oligodendroglial cells and are expressed in this cell lineage at all times. They include the Olig proteins as members of the bHLH transcription factor family, in particular, Olig2, as well as the HMG domain transcription factor Sox10 [17,19,20]. Sox10 in particular has been shown to influence various sets of genes during different stages of oligodendroglial development, including other transcription factors, components of signaling pathways, myelin genes, and microRNAs [21,22,23].

When analyzing deregulated gene expression in oligodendroglial cells upon Sox10 inactivation, we noticed a strong and significant downregulation of several voltage-gated ion channels that have already been implicated in oligodendroglial development or physiology. Therefore, we set out to determine whether the genes coding for these ion channels are under direct transcriptional control of Sox10 and represent another group of target genes by which Sox10 exerts its impact on oligodendroglial development and differentiation.

## 2. Materials and Methods

### 2.1. Plasmids

The pCMV5-based expression plasmid for Sox10 has been described before [23]. Luciferase reporter plasmids containing potential regulatory regions from the *Scn1a*, *Cacna1b*, *Kcna1*, and *Kcnj10* locus were generated by amplifying the respective sequences from mouse embryonic stem cell DNA by PCR and inserting them (in case of promoters) alone or (in case of potential enhancers) in combination with a β-globin minimal promoter into the multiple cloning site of pGL2-luc (Promega, Walldorf, Germany). Sequences included: *Scn1a* prom (mm10, chr2:66,409,975–66,411,074, 1100 bp), *Scn1a* +68RR (mm10, chr2:66,341,011–66,342,737, 1727 bp), *Scn1a* +139RR (mm10, chr2:66,269,364–66,270,995, 1632 bp), *Scn1a* +145RR (chr2:66,262,130–66,263,128, 999 bp), *Cacna1b* prom (mm10, chr2:24,763,108–24,764,081, 974 bp), *Cacna1b*-4RR (mm10, chr2:24,766,988–24,768,248, 1261 bp), *Cacna1b* +78RR (mm10, chr2:24,682,452–24,683,613, 1162 bp), *Cacna1b* +79RR (mm10, chr2:24,683,571–24,684,947, 1377 bp), *Cacna1b* +83RR (mm10, chr2:24,679,833–24,680,832, 1000 bp), *Kcna1* prom (mm10, chr6:126,644,735–126,646,117, 1383 bp), *Kcna1* -40RR (mm10, chr6:126,684,694–126,685,871, 1178 bp), *Kcnj10* prom (mm10, chr1:172,340,109–172,341,209, 1101 bp), and *Kcnj10* +24RR (mm10, chr1:172,349,548–172,350,895, 1348 bp).

### 2.2. Cell Culture, Luciferase Assays

Neuro2a (ATCC CCl-131) and Oln93 cells [24] were maintained in high glucose DMEM (Thermo Fisher Scientific, Dreieich, Germany, Gibco 11965092) supplied with 10% FCS (Thermo Fisher Scientific, Dreieich, Germany, Gibco 10500064) and Pen/Strep (Thermo Fisher Scientific, Dreieich, Germany, Gibco 15140122). CRISPR/Cas9-mediated Sox10 inactivation in Oln93 cells has been described before [22]. To restore the expression of Sox10, Sox10-deleted Oln93 cells were transduced by a Sox10- and GFP-encoding lentivirus at a moiety of infection of 1 and purified by fluorescence-activated cell sorting. Analogous transductions were carried out with Sox8- and Sox9-expressing lentiviruses.

For luciferase reporter assays, Neuro2a cells were seeded in 24-well plates and transfected per well with 200 ng of luciferase reporter plasmid and 25 ng, 100 ng, or 250 ng (+, ++, +++ in Figures 2–4) of pCMV5-based expression plasmids using Xfect (Takara Biotech, Saint-Germain-en-Laye, France, 631318) according to the manufacturer’s instructions. Overall DNA amounts per well were kept constant. Luciferase assays and their analysis have been reported before [21,25]. Primary oligodendroglial progenitor cells (OPCs) were obtained from dissected forebrains of newborn Wistar rats or E18.5 transgenic and control mice via mixed glial cultures and subsequent shake-off and microglial depletion [26,27]. Primary rat oligodendroglial cells were seeded on poly-ornithine-coated (Sigma-Aldrich, Hamburg, Germany, P3655) cell culture dishes and cultured either in basal medium supplied with PDGF-AA (Thermo Fisher Scientific, PeproTech, cat#100-13A) and bFGF (Thermo Fisher Scientific, PeproTech, cat#100-18B) for proliferation or differentiated by replacing growth medium with serum-free SATO medium (containing high T3/T4 levels) supplemented with 20 pg/mL of CNTF (Thermo Fisher Scientific, PeproTech, cat#450-50) for up to 3 days as described before [25]. Primary mouse oligodendroglial cells were seeded on poly-d-lysine (Sigma-Aldrich, Hamburg, Germany, P64407) and laminin-coated (Sigma-Aldrich, Hamburg, Germany, L2020) dishes and cultured in basal medium under the same proliferative conditions as rat oligodendroglial cells for 1 day.

### 2.3. Quantitative RT-PCR

RNA was prepared under proliferating and differentiating conditions from cultured primary rodent oligodendroglial cells and from Oln93 cells using Trizol (Thermo Fisher Scientific, Invitrogen, 15596026). Reverse transcription into cDNA was performed as previously described [25]. Samples were then used for quantitative PCR on a Biorad CFX96 Real-Time PCR System using PowerUp SYBR Green Mastermix (Thermo Fisher Scientific). The following primers were used: 5′-CTTCCCCACATCAGTCTCTGT-3′ and 5′-CCAGCATTCGGGAGGATCTG-3′ for *Scn1a*, 5′-AGACCATCAAGCGGCTGCCTAA-3′ and 5′-TGAAGAGCTGGACGGCAATGAC-3′ for *Cacna1b*, 5′-GAGTCGCACTTCTCCAGTATCC-3′ and 5′-CCCACGATCTTGCCTCCAATTG-3′ for *Kcna1*, and 5′-TGCGGAAGAGTCTCCTCATTGG-3′ and 5′-GTCTGAGGCTGTGTCTACTTGG-3′ for *Kcnj10*. Primers for *Gapdh* and *Rpl8* were as described [25]. All samples were processed as technical triplicates. The melting curve of each sample was measured to ensure the specificity of the amplified products. Data were analyzed by the ΔΔCt method [28] and normalized to *Gapdh* and *Rpl8* expression.

### 2.4. Chromatin Immunoprecipitation (ChIP)

Sheared chromatin was precipitated with self-made rabbit anti-Sox10 antiserum [29] and corresponding rabbit pre-immune serum coupled to BSA-blocked Protein A-Sepharose beads (GE Healthcare, Düsseldorf, Germany, 17–0780-01) as published [25]. After purification from input and precipitated chromatin fragments, DNA was used for quantitative PCR to calculate the percent recovery of specific DNA regions from the total input with protein-specific antibodies in relation to corresponding control antibodies using the ΔΔCt method. The following primers were used: 5′-TATAGTGGACGGCTCTGAAGGA-3′ and 5′-TCATGCGTGACCAAATGTGA-3′ for *Scn1a* prom, 5′-TGTGGCAAACTTAATAGTTCCAAAG-3′ and 5′-CTGCAGTCTATAATTGTATGCAACC-3′ for *Scn1a* +145RR, 5′-GCCTAGGTTCTCATTGGCGT-3′ and 5′-CAAACAGGTGCACCACGAAG-3′ for *Cacna1b* prom, 5′-AAGAGCATCGCTTCACACCA-3′ and 5′-TCCTGGTGATGAAAGGACCC-3′ for *Cacna1b* +79RR, 5′-GTGCTGTTCAGATGGCGACT-3′ and 5′-GCAAGTGATGTCGCTTTTGC-3′ for *Kcna1* -40RR, 5′-CCTCGGGGAGGGTACATAGT-3′ and 5′-AAGCCAGAGAACGGTGCAAA-3′ for *Kcnj10* prom, 5′-GTCACCTGCTTTCAGAGCCT-3′ and 5′-TACCCTTTTCTGTCACGGGC-3′ for *Kcnj10* +24RR, 5′-GCCTAGCACCAACCGATACA-3′ and 5′-ATGGTATTGGTCCAGCAAGGG-3′ for *Scn1a* +68RR neg, and 5′-CACTGGGATTAGATCTGAGCC-3′ and 5′-CTGACCTGAAGAGATTCCTGC-3′ for *Cspg4* neg.

### 2.5. Husbandry and Breeding of Mice

Transgenic and control mice were on a mixed C3H × C57Bl/6J background and housed under standard conditions with 12 h dark–light cycles and continuous access to food and water according to animal welfare laws and relevant ethical regulations. Both male and female mice with relevant genotypes were used for analysis. Oligodendroglial knockout of Sox10 was achieved by crossing *Sox10^fl/fl^* mice [30] with *Olig2-Cre* mice [31] for immunohistochemical studies (Sox10ΔOL mice) or *Sox10-Cre* mice [32] to generate *Sox10^−/−^* primary mouse oligodendroglial cells. Genotyping was as described [30,31,32]. Timed matings were set up to generate litters at embryonic day (E) 18.5 and postnatal day (P) 7 for preparation of spinal cord tissue or primary mouse oligodendroglial cell cultures.

### 2.6. In Situ Hybridization and Immunohistochemistry

After fixation of spinal cord tissue in 4% paraformaldehyde, dehydration in 30% sucrose, and freezing, sections were generated on a Leica cryotome (Leica, Wetzlar, Germany). Immunohistochemistry and in situ hybridization were performed on 10 µm transverse spinal cord sections (forelimb level). In situ hybridization was performed with DIG-labeled antisense riboprobes specific for *Kcnj10* (nucleotides 991 to 1777 of NM_001039484.1). For immunohistochemistry, the following primary antibodies were used: goat anti-Pdgfra (R&D systems, Wiesbaden, Germany, #AF1062, Lot #HMQ0222061, 1:50 dilution), rabbit anti-Na_v_1.1 (Alomone Labs, Jerusalem, Israel #ASC-001, Lot #AN3702/YE3934594, 1:400 dilution), rabbit anti-Ca_v_2.2 (Alomone Labs, #ACC-002, Lot #AN4402/YE3933311, 1:200 dilution), and rabbit anti-K_v_1.1 (Alomone Labs, #APC-009, Lot #AN1002/YE3933315, 1:100 dilution) antibodies. Secondary antibodies were coupled to Cy3 (Dianova, Hamburg, Germany 1:200 dilution), Cy5 (Dianova, 1:200 dilution), or Alexa Fluor 488 (Molecular Probes, Eugene, OR, USA, 1:500 dilution) fluorescent dyes. Nuclei were counterstained with 4’,6-diamidino-2-phenylindole dihydrochloride (DAPI). The staining results were documented on a camera-equipped Leica MZFLIII stereomicroscope or Leica DMI6000 B inverted microscope (Leica).

### 2.7. Statistical Analysis

Biological replicates were defined as results from independent animals, experiments, or separately and independently generated samples. The sample size was *n* ≥ 3 for all mice and molecular biology experiments. Investigators were not blinded in animal experiments. GraphPad Prism version number 6 (GraphPad software, version 6.0, La Jolla, CA, USA) was used for statistical testing using two-tailed Student’s *t*-tests and analysis of variance (ANOVA) to determine differences in cell numbers, luciferase activity, transcript levels, or immunoprecipitated DNA (* *p* ≤ 0.05, ** *p* ≤ 0.01, *** *p*  ≤  0.001). The variance between statistically compared groups was similar.

## 3. Results

### 3.1. Voltage-Gated Ion Channels Are Downregulated upon Sox10 Inactivation in an Oligodendroglial Cell Line

**Figure 1:**
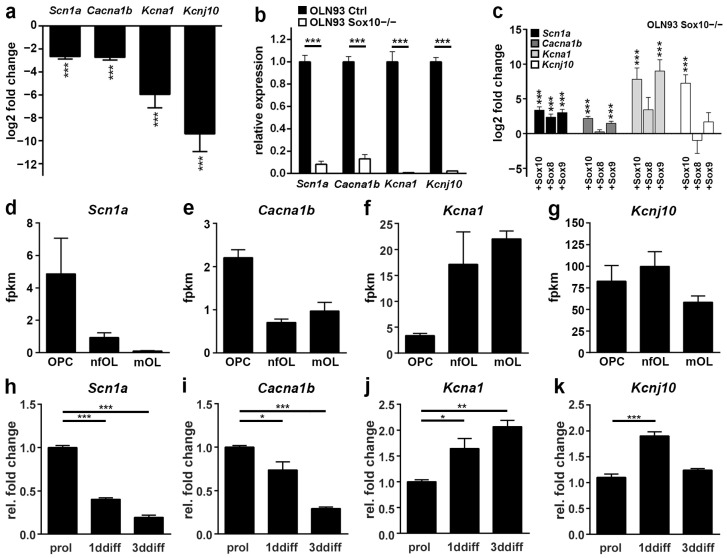
Analysis of the expression of voltage-gated ion channels in oligodendroglial cells. (**a**,**b**) Altered expression of *Scn1a*, *Cacna1b*, *Kcna1*, and *Kcnj10* in Oln93 cells upon Sox10 inactivation according to previously published RNA-seq data [(**a**), GSE136659] or quantitative RT-PCR (**b**) on control (ctrl) and Sox10-deficient (Sox10^−/−^) Oln93 cells. (**c**) Changes in *Scn1a*, *Cacna1b*, *Kcna1*, and *Kcnj10* expression induced in Sox10-deficient Oln93 cells upon lentiviral transduction of Sox10 or its close relatives Sox8 and Sox9 as determined by quantitative RT-PCR. (**d**–**g**) Comparative expression of *Scn1a* (**d**), *Cacna1b* (**e**), *Kcna1* (**f**), and *Kcnj10* (**g**) in OPCs and newly formed and myelinating oligodendrocytes (nfOL and mOL) purified from the neonatal brain according to RNA-seq data [33]. (**h**–**k**) Expression of *Scn1a* (**h**), *Cacna1b* (**i**), *Kcna1* (**j**), and *Kcnj10* (**k**) in primary oligodendroglial cells kept under proliferative (prol) or differentiating conditions for 1–3 days (1ddiff, 3ddiff) as determined by quantitative RT-PCR. Data represent mean values ± SEM from single sequencing runs (**a**,**c**,**d**–**g**) or RT-PCR on separate RNA samples (**b**,**h**–**k**; *n* ≥ 3) and were statistically analyzed by Student’s *t*-test (**b**) and one-way ANOVA with Bonferroni correction (**h**–**k**), respectively (* *p* ≤ 0.05, ** *p* ≤ 0.01, *** *p* ≤ 0.001).

Oln93 cells arose by spontaneous immortalization of rat oligodendrocyte progenitor cells (OPCs) and represent one of the few available oligodendroglial cell culture models [24]. We previously inactivated the oligodendroglial transcription factor Sox10 in these cells by CRISPR/Cas9-dependent genome editing [22] and analyzed the resulting changes in the expression profile by RNA-seq analysis [21]. By analyzing these previously published data, several voltage-gated ion channels were found to be significantly downregulated in Sox10-deficient Oln93 cells (Figure 1a). These included the tetrodotoxin-sensitive sodium channel Na_v_1.1 (encoded by the *Scn1a* gene), the N-type calcium-channel Ca_v_2.2 (encoded by the *Cacna1b* gene), the outward-rectifying potassium channel K_v_1.1 (encoded by the *Kcna1* gene) and the inward-rectifying potassium channel Kir4.1 (encoded by the *Kcnj10* gene). Quantitative RT-PCR on Oln93 cells with and without Sox10 confirmed the strongly reduced expression of all four genes in the absence of Sox10 (Figure 1b). Intriguingly, restoration of Sox10 expression by lentiviral transduction increased transcript levels for all four voltage-gated ion channels in Sox10-deficient Oln93 cells (Figure 1c). Lentiviral transduction of the closely related Sox9 had a similar stimulatory effect on ion channel gene expression, whereas transduction of the third close relative Sox8 only increased *Scn1a* and *Kcna1*, but not *Cacna1b* and *Kcnj10* transcript levels. This argues that the three paralogs have similar but not identical effects on the expression of these ion channel genes.

A further look at published RNA-seq data sets (https://brainrnaseq.org, last accessed on 30 May 2024) revealed that the four voltage-gated ion channels exhibit different temporal expression patterns, with *Scn1a* and *Cacna1b* transcript levels being high in OPCs and declining as oligodendrocytes differentiate, and *Kcna1* levels reciprocally increasing from the OPC stage to myelinating oligodendrocytes (Figure 1d–f). In contrast, *Kcnj10* levels are high in OPCs and differentiating oligodendrocytes and remain comparable among all stages (Figure 1g). The *Kcnj10* expression pattern is most similar to the one of Sox10, where high levels of transcripts and protein are detected at all stages from OPCs to myelinating oligodendrocytes [17,21,23]. Matching expression profiles were obtained for the voltage-gated ion channels in primary cultures of oligodendroglial cells, kept either under proliferative conditions or in differentiation media for 1 to 3 days (Figure 1h–k). We conclude from these data that the expression of the ion channel genes depends on the presence of Sox10 independent of their respective expression profile during oligodendroglial development.

### 3.2. The Scn1a Promoter and a Putative Regulatory Region at +145 kb Are Transcriptionally Activated by Sox10

**Figure 2:**
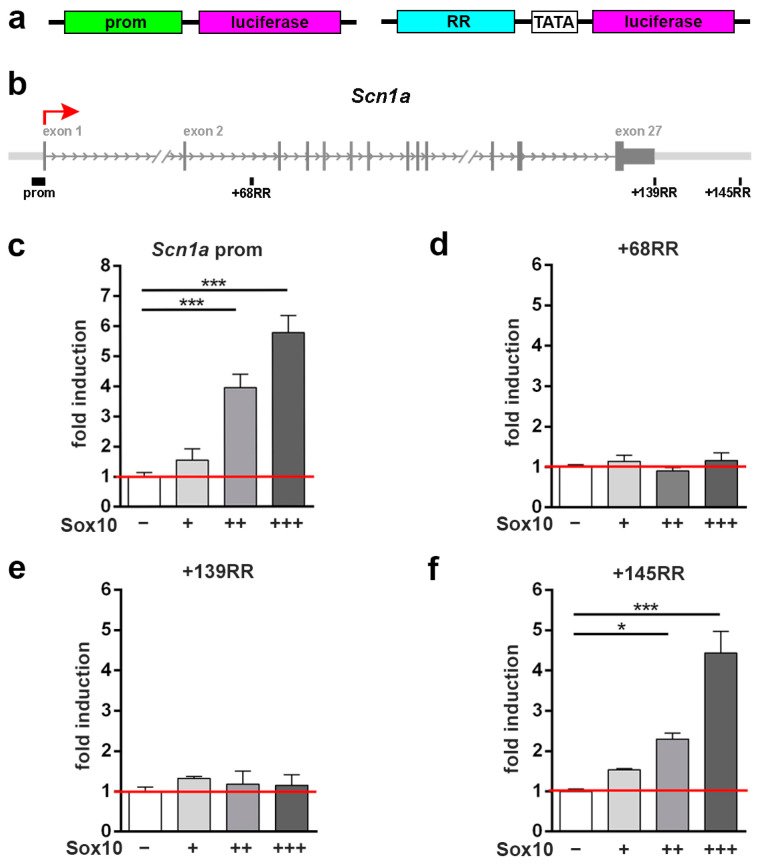
Analysis of Sox10-responsive regions in the *Scna1* locus. (**a**) Design of luciferase reporters for the analysis of gene promoters (prom, left) and other potential regulatory regions (RR, right). (**b**) Scheme of the *Scna1* genomic locus. Shown are introns (arrows) and exons (vertical bars) of the *Scna1* gene as well as the position of the promoter, the RRs, and the transcriptional start site (red arrow). (**c**–**f**) Luciferase assays in Neuro2a cells transiently transfected with reporter genes under the control of the promoter (**c**) as well as the +68RR (**d**), +139RR (**e**), and +145RR (**f**) of the *Scna1* gene in the absence (−) or presence of increasing amounts (+, ++, +++) of Sox10 as indicated below the bars. Effector-dependent activation rates are presented as fold inductions ± SEM with transfections in the absence of Sox10 set to 1 (*n* ≥ 3). Differences were statistically significant as determined by one-way ANOVA with Bonferroni correction (* *p* ≤ 0.05, *** *p* ≤ 0.001).

As expression of the voltage-gated ion channel genes changed with the presence of Sox10, it appeared possible that the genes were direct downstream targets of this transcription factor. To study this possibility in further detail, we analyzed the region surrounding the four genes for the presence of putative Sox10-binding regions according to previously published ChIP-seq studies [34] and/or strong evolutionary conservation. The potential regulatory regions (RRs) and the gene promoter were inserted into luciferase reporter plasmids (Figure 2a) and analyzed in transiently transfected Neuro2a cells for their responsiveness towards Sox10.

For the *Scn1a* gene, three potential regulatory regions at +68 kb (+68RR) in intron 2 and at +139 kb (+139RR) and +145 kb (+145RR) immediately behind the gene were identified and tested in addition to the promoter (Figure 2b). While the *Scn1a* promoter became more active in the presence of Sox10 in a dose-dependent manner, the intronic +68RR proved unresponsive even at the highest Sox10 amount tested (Figure 2c,d). Among the downstream regions only, +145RR, but not +139RR, exhibited an increased activity upon co-transfection of Sox10 (Figure 2e,f). We conclude from these data that both the promoter and the +145RR are Sox10-responsive and may mediate a Sox10-dependent activation of the *Scn1a* gene.

### 3.3. The Cacna1b Promoter and a Putative Regulatory Region at +78 kb Are Transcriptionally Activated by Sox10

**Figure 3:**
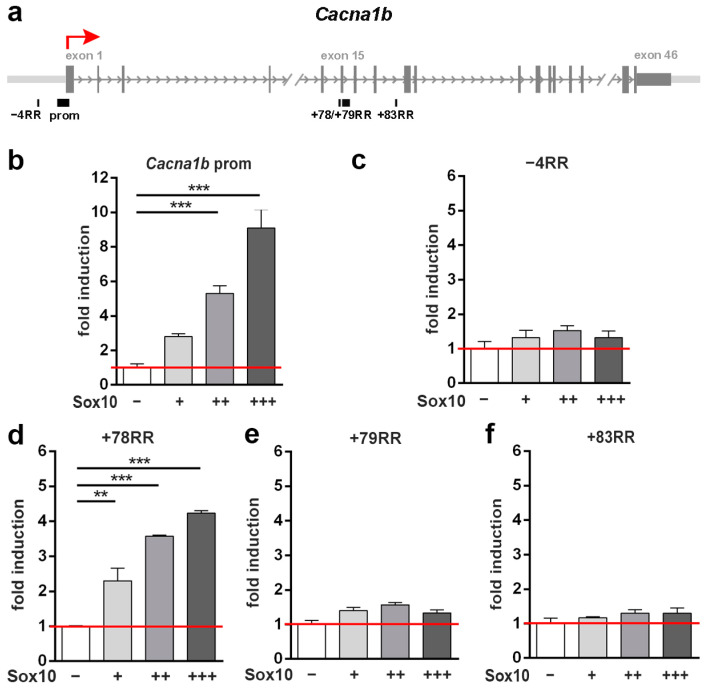
Analysis of Sox10-responsive regions in the *Cacna1b* locus. (**a**) Scheme of the genomic locus. Shown are introns (arrows) and exons (vertical bars) of the *Cacna1b* gene as well as the position of the promoter, the RRs, and the transcriptional start site (red arrow). (**b**–**f**) Luciferase assays in Neuro2a cells transiently transfected with reporter genes under the control of the promoter (**b**) as well as the −4RR (**c**), +78RR (**d**), +79RR (**e**), and +83RR (**f**) of the *Cacna1b* gene in the absence (−) or presence of increasing amounts (+, ++, +++) of Sox10 as indicated below the bars. Activation rates are shown as fold inductions ± SEM with transfections in the absence of Sox10 set to 1 (*n* ≥ 3). Differences were statistically significant as determined by one-way ANOVA with Bonferroni correction (** *p* ≤ 0.01, *** *p* ≤ 0.001).

In the case of the *Cacna1b* gene, we identified four RRs in addition to the promoter. These RRs were distributed over 90 kb, starting with a RR at −4 kb in front of the gene (−4RR) to RRs at +78 kb, +79 kb, and +83 kb (+78RR, +79RR, and +83RR) within the gene body (Figure 3a). Upon insertion into luciferase reporters and transfection into Neuro2a cells, the activity of the promoter was robustly increased by co-transfected Sox10 (Figure 3b). Among the other RR, Sox10-dependent stimulation was only observed for the +78RR (Figure 3d). All the other RRs did not exhibit a significantly altered activity in the presence of Sox10 over a wide range of concentrations (Figure 3c,e,f).

### 3.4. A Putative Regulatory Region 40 kb in Front of the Kcna1 Gene Is Transcriptionally Activated by Sox10

**Figure 4:**
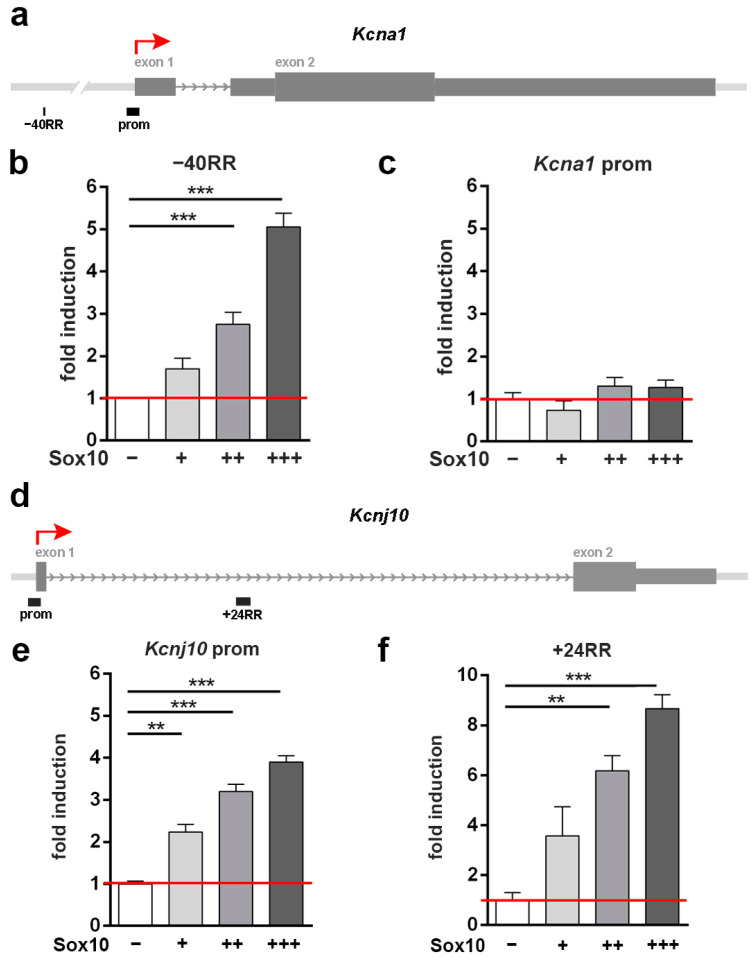
Analysis of Sox10-responsive regions in the *Kcna1* and *Kcnj10* loci. (**a**,**d**) Scheme of the genomic loci. Shown are introns (arrows) and exons (boxes) of the *Kcna1* (**a**) and *Kcnj10* (**d**) genes as well as the position of the promoter, the RRs, and the transcriptional start site (red arrow). (**b**,**c**,**e**,**f**) Luciferase assays in Neuro2a cells transiently transfected with reporter genes under the control of the -40RR (**b**) or the promoter (**c**) of the *Kcna1* gene as well as the promoter (**e**) or +24RR (**f**) of the *Kcnj10* gene in the absence (−) or presence of increasing amounts (+, ++, +++) of Sox10 as indicated below the bars. Activation rates are shown as fold inductions ± SEM with transfections in the absence of Sox10 set to 1 (*n* ≥ 3). Differences were statistically significant as determined by one-way ANOVA with Bonferroni correction (** *p* ≤ 0.01, *** *p* ≤ 0.001).

In contrast to the *Scn1a* and *Cacna1b* genes, only one RR was identified for the *Kcna1* gene. This RR was localized at −40 kb relative to the transcriptional start site (Figure 4a) and responded to Sox10 in co-transfection experiments with increasing activity in a dose-dependent manner (Figure 4b). The *Kcna1* promoter, in contrast, was refractory to the presence of Sox10 (Figure 4c).

### 3.5. The Kcnj10 Promoter and a Putative Regulatory Region at +24 kb Are Transcriptionally Activated by Sox10

For *Kcnj10*, we again identified a single RR (+24RR) within the large first intron of the gene, approximately 24 kb behind the transcriptional start site (Figure 4d). Both the *Kcnj10* promoter and the +24RR increased their activity significantly upon co-transfection of Sox10 in Neuro2a cells (Figure 4e,f). We conclude from our reporter gene assays that all four voltage-gated ion channel genes contain one or more regions that respond to the presence of Sox10 with increased transcriptional activity in reporter gene assays. For three of the four genes, these regions include the gene promoter. However, responsive regions are not restricted to promoters for any of the tested genes.

### 3.6. Regulatory Regions of Voltage-Gated Ion Channel Genes Are Bound by Sox10 In Vivo

**Figure 5:**
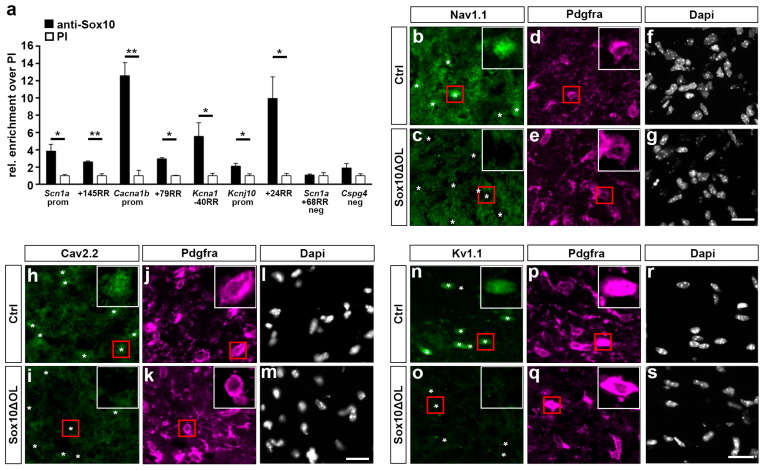
Influence of Sox10 on voltage-gated ion channel occurrence in oligodendroglial cells in vivo. (**a**) ChIP experiments on wildtype CG4 cells. Chromatin was precipitated with a Sox10 antiserum and the corresponding pre-immune serum to detect Sox10 occupancy on promoters and RRs from the *Scn1a*, *Cacna1b*, *Kcna1*, and *Kcnj10* genes and a negative control region from the *Cspg4* gene. Precipitated chromatin was normalized to input and is shown as relative fold enrichment with amounts obtained for pre-immune serum set to 1. Data represent mean values (*n* ≥ 3) ± SEM and were statistically analyzed by Student’s two-tailed *t*-test (* *p* ≤ 0.05, ** *p* ≤ 0.01. (**b**–**s**) Immunohistochemical staining of spinal cord tissue from control (**b**,**d**,**f**,**h**,**j**,**l**,**n**,**p**,**r**) and Sox10ΔOL (**c**,**e**,**g**,**i**,**k**,**m**,**o**,**q**,**s**) mice at E18.5 using antibodies directed against Na_v_1.1 (**b**,**c**), Ca_v_2.2 (**h**,**i**), and K_v_1.1 (**n**,**o**) in combination with antibodies directed against Pdgfra (**d**,**e**,**j**,**k**,**p**,**q**) and Dapi (**f**,**g**,**l**,**m**,**r**,**s**). Pdgfra-expressing oligodendroglial cells are marked in **b**,**c**,**h**,**i**,**n**,**o** by white asterisks in the panels with the ion channel immunostaining. Red boxes in panels mark the position of a representative Pdgfra-positive OPC. The same OPC is shown magnified in the right upper corner (inlay, white box). Scale bars: 20 µm.

To investigate whether the Sox10-responsive promoters and RRs would also be occupied by Sox10 in vivo, we analyzed all regions in chromatin immunoprecipitation experiments. For these experiments, we precipitated chromatin from Sox10-positive oligodendroglial CG4 cells with Sox10-specific and pre-immune antisera and determined by quantitative PCR whether Sox10-responsive promoters and RRs from the *Scna1*, *Cacna1b*, *Kcna1*, and *Kcnj10* genes would be enriched in the precipitates obtained with Sox10-specific antibodies relative to pre-immune controls. As shown in Figure 5a, this was indeed the case, although enrichment over the controls varied substantially for the Sox10-responsive regions. At the same time, other regions from the *Scn1a* or *Cspg4* gene loci that were unresponsive towards Sox10 in luciferase assays did not exhibit a comparable enrichment (Figure 5a). These results provide additional evidence for a direct effector–target gene relationship between Sox10 and the voltage-gated ion channels in oligodendroglial cells.

### 3.7. Oligodendroglial Expression of Voltage-Gated Ion Channel Genes Is Severely Affected In Vivo following Sox10 Inactivation

If *Scna1*, *Cacna1b*, *Kcna1*, and *Kcnj10* are direct target genes of Sox10, their expression should be substantially affected in mice that overexpress Sox10 in oligodendroglial cells or carry an oligodendroglial Sox10 deletion. We decided on the loss-of-function approach because of the availability of the mouse model and the higher physiological relevance. While we did not study the consequences of Sox10 overexpression on voltage-gated ion channel expression in vivo, we generated mice in which Sox10 is deleted in oligodendroglial cells by combining *Sox10^fl^* alleles with a Cre allele (*Olig2-Cre* or *Sox10-Cre*) that becomes active early in OPCs (Sox10ΔOL). When spinal cord tissue from these mice was analyzed on transverse embryo sections at E18.5 by immunohistochemistry with a Na_v_1.1 antibody, we observed a reduction in the overall signal intensity in Sox10ΔOL mice compared to the controls (Figure 5b,c). A closer look at Pdgfra-positive OPCs as the oligodendroglial cells with the highest Na_v_1.1 expression further revealed that the Nav1.1 signal was preferentially lost in these cells (Figure 5d–g). A similar overall reduction in staining intensity and a selective loss of expression was also observed in Sox10ΔOL mice when spinal cord tissue was probed with antibodies directed against Ca_v_2.2 (Figure 5h–m) or K_v_1.1 (Figure 5n–s). These results support the notion that the expression of Na_v_1.1, Ca_v_2.2, and K_v_1.1 in oligodendroglial cells requires the presence of Sox10.

**Figure 6:**
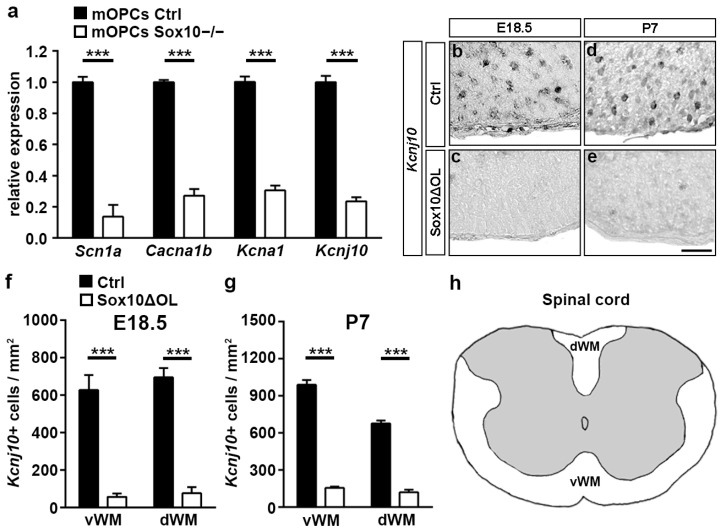
Influence of Sox10 on voltage-gated ion channel occurrence in oligodendroglial cells in vivo. (**a**) Quantification of *Scn1a*, *Cacna1b*, *Kcna1*, and *Kcnj10* transcript levels by quantitative RT-PCR in cultures of primary mouse oligodendroglial cells obtained from Sox10ΔOL and control mice. Data represent mean values ± SEM from separate samples (*n* = 3) with mean values from the controls set to 1. (**b**–**h**) In situ hybridization of spinal cord tissue from the control (**b**,**d**) and Sox10ΔOL (**c**,**e**) mice at E18.5 (**b**,**c**) and P7 (**d**,**e**) using an antisense riboprobe specific for *Kcnj10*, and resulting quantifications (**f**,**g**) of *Kcnj10*-positive cells in ventral white matter (vWM) and dorsal white matter (dWM, for scheme see panel **h**) presented as mean values ± SEM (*n* = 3). Scale bar: 100 µm. Statistical significance was determined by Student’s two-tailed *t*-test (*** *p* ≤ 0.001).

Additional expression of these voltage-gated ion channels in other cells of the spinal cord, their widespread distribution through various cellular compartments, and the low expression levels of most of them limit the interpretability of the immunohistochemical results. For Kir4.1, we failed to obtain meaningful staining results altogether. Therefore, we additionally generated cultures of primary mouse oligodendroglial cells from Sox10ΔOL and control mice at E18.5 and analyzed RNA from these cells for expression of the voltage-gated ion channels by quantitative RT-PCR. These analyses confirmed dramatically lower levels of *Scna1*, *Cacna1b*, and *Kcna1* transcript levels in Sox10-deficient cells than in the control oligodendroglial cells (Figure 6a). It also pointed to a comparable downregulation of *Kcnj10* transcripts upon Sox10 loss. To confirm the importance of Sox10 for *Kcnj10* expression, we carried out in situ hybridization on spinal cord tissue of Sox10ΔOL and control mice at E18.5 and P7 (Figure 6b–e). Quantification of the staining results revealed a drastic reduction in *Kcnj10*-expressing (presumably oligodendroglial) cells in both dorsal and ventral white matter regions of Sox10ΔOL mice at both time points. *Kcnj10*-expressing cells were reduced to 9–17% at E18.5 and P7 in both white matter regions compared to the wildtype (Figure 6f–h). The number of *Kcnj10*-expressing (mostly neuronal) cells in the grey matter remained unaltered. Taken together, the expression of all four voltage-gated ion channels is strongly reduced in oligodendroglial cells upon Sox10 loss, supporting a requirement of Sox10 for their expression. Interestingly, ion channel expression is reduced in Sox10-deficient oligodendroglial cells despite the continued presence of the partially redundant Sox8. This observation correlates well with our finding that voltage-gated ion channel expression is poorly rescued by Sox8 in Sox10-deficient Oln93 cells (see Figure 1c). The molecular reason for this functional difference between the paralogs is currently unknown.

## 4. Discussion

Here we provide in vitro and in vivo evidence that several voltage-gated ion channels with occurrence and function in oligodendroglial cells are directly regulated in their expression by the Sox10 transcription factor. We show that each of the analyzed genes for voltage-gated ion channels contains at least one Sox10-responsive region such as the respective gene promoters or other regulatory elements. These Sox10-responsive regions are furthermore all bound by Sox10 in vivo according to chromatin immunoprecipitation data, independent of their localization in front, behind, or within the gene. Additionally, expression of the voltage-gated ion channels decreases upon loss of Sox10 in oligodendroglial cells both in culture and in situ in mouse CNS tissue. At least in cell culture, the reintroduction of Sox10 rescues the expression. Considering the fact that the studied voltage-gated ion channels are known to endow OPCs with the ability to fire action potentials or allow mature oligodendrocytes to remove extracellular K^+^ to ensure axonal function and integrity [2,5,6,7,8,9,10,13,14], it seems likely that Sox10 exerts some of its effects in oligodendroglial cells via these voltage-gated ion channels. Our study also links voltage-gated ion channels to the transcriptional regulatory network and, thus, joins groups of proteins that are usually analyzed and treated separately.

Among the analyzed ion channels, Na_v_1.1 and Ca_v_2.2 are preferentially expressed in OPCs, where only a handful of Sox10 target genes are known, among them *Pdgfra* and *Cspg4* [21,35,36]. Our current study therefore substantially increases the known target genes and downstream effectors of Sox10 in OPCs. In contrast, K_v_1.1 amounts increase in differentiating oligodendrocytes, whereas Kir4.1 levels remain fairly unchanged during oligodendroglial development. Voltage-gated ion channels therefore nicely illustrate that Sox10 can directly activate the expression of genes preferentially expressed in OPCs or genes that mostly occur in oligodendrocytes in addition to genes that exhibit expression throughout the whole lineage [21]. How a single, continuously expressed transcription factor can obtain such stage-specificity that furthermore varies between genes is probably multifactorial. One important way of achieving this has been described and involves the synergistic and antagonistic interactions of Sox10 with other transcription factors that exhibit a stage-specific expression pattern during oligodendroglial development (e.g., [21,37]. We would therefore expect that the regulatory regions identified in this study all contain Sox10-binding sites, but will differ in the binding sites for other transcription factors so that they form different classes of Sox10-responsive enhancers with different temporal activities. Our current analysis lacks the resolution to map the relevant Sox10-binding sites within the regulatory regions at the nucleotide level and has not addressed the role of possible interaction partners. Therefore, the exact architecture of the regulatory regions and related questions, such as whether there are different enhancer classes that conform to a general code, remain to be experimentally addressed in future studies.

Most of the genes for the studied voltage-gated ion channels are fairly large and exhibit expression in various cell types of the CNS, including many neurons and astrocytes. Considering that Sox10 expression in the CNS is restricted to oligodendroglial cells and that even its closest relatives, Sox8 and Sox9, are not widely expressed in neurons, it is likely that the mechanism of activation of ion channel gene expression described in this study is only valid in oligodendroglial cells. We therefore conclude that other transcription factors are involved in the activation of ion channel gene expression in neurons (and astrocytes) and that the regulatory code differs between cell types. Unfortunately, transcriptional regulation of ion channels is not only poorly studied in oligodendroglial cells but also in neurons [38,39]. In theory, it is possible that the same regulatory regions are targeted by different sets of transcription factors. In agreement with the current literature, however, we favor a model in which separate sets of regulatory regions drive specific expression in the various CNS cell types. Whether this is indeed the case, is again subject to further analysis.

## 5. Conclusions

Despite the limitations of this study, we have clearly shown that Sox10 activates several relevant voltage-gated ion channels in oligodendroglial cells so that Sox10 activity at various stages of oligodendrocyte development may be linked to channel function. These take-home messages are not only relevant for developmental myelination and myelin maintenance but also for remyelination after injury or other demyelinating events.

## Data Availability

All data generated or analyzed during this study are included in this published article.
